# What are the active ingredients of ‘arts in health’ activities? Development of the INgredients iN ArTs in hEalth (INNATE) Framework

**DOI:** 10.12688/wellcomeopenres.17414.1

**Published:** 2022-01-11

**Authors:** Katey Warran, Alexandra Burton, Daisy Fancourt

**Affiliations:** 1 Research Department of Behavioural Science and Health, Institute of Epidemiology & Health Care, University College London, 1-19 Torrington Place, London, WC1E 7HB, UK

**Keywords:** Active ingredients, components, arts and culture, arts interventions, arts in health

## Abstract

Background: There is a scarcity of research concerning what it is about arts engagement that may activate causal mechanisms leading to effects on health and wellbeing outcomes: their active ingredients. Further, the limited studies that do exist have tended to be relevant to specific contexts and types of art forms. The aim of this study was to carry out a comprehensive mapping of potential active ingredients, construct a shared language, and propose a framework and toolkit to support the design, implementation, and evaluation of arts in health activities.

Methods: Drawing upon Rapid Appraisal techniques and collaborating with 64 participants, we engaged in a three-phase process: 1) a scoping review to inform the development of an initial framework; 2) consultation on the initial framework; and 3) analysis and construction of the INNATE framework.

Results: The study identified 139 potential active ingredients within the overarching categories of *project, people, *and *contexts*. *Project *components relate directly to the content of the arts activity itself, intrinsic to what the activity is. The *people *category denotes how people interact through engagement with the activity and who is involved in this interaction, including activity facilitation. *Contexts *relates to the activity setting comprising the aggregate of place(s), things, and surroundings. Aligning with complexity science, Ingredients may overlap, interconnect, or feed into one another to prompt mechanisms, and may not be experienced as distinct by participants.

Conclusions: Our mapping exercise is the most extensive to date. In relation to arts in health activities, the INNATE framework can support with: design and implementation, such as co-producing an intervention to meet the needs of a particular population; evaluation, such as facilitating the comparison of different interventions and their efficacy; and replication, scalability, and sustainability through enabling detailed reporting and specific articulation of what an arts in health activity entails.

## Background

Over the last 20–25 years there has been a rapid increase in research exploring the benefits of arts and cultural activities for health and wellbeing ^1, 2^. The research conducted highlights the impact that these activities can have in supporting with the management and treatment of mental and physical health conditions, as well as preventing ill health and increasing health promoting behaviours ^2^. In seeking to understand how this impact is achieved, there has been increasing interest in understanding the mechanisms of action behind these impacts. ^1^ Over 600 mechanisms have been identified, including those that involve psychological, biological, social, and behavioural processes ^3^. What remains less clear is what it is about arts and culture that triggers these mechanisms: what the components or active ingredients are that initiate the mechanisms of action that, in turn, affect mental and physical health outcomes.

### Active ingredients

The term ‘active ingredients’ has its origins in pharmacological research for describing the elements of a pharmacologic intervention responsible for its therapeutic action ^4^, and is often used interchangeably with ‘intervention components’ or ‘interacting components’ ^5, 6^. Over the past two decades, research into ‘active ingredients’ has also become increasingly popular within non-pharmacological health research seeking to identify the ‘what’ rather than the ‘how’ of interventions ^7^. Much of this non-pharmacological work on active ingredients has occurred within the fields of behavioural science (which focuses on human behaviours) and implementation science (which focuses on promoting ‘the systematic uptake of research findings and other evidence-based practices into routine practice’ ^8^). Such work has involved the identification of active ingredients involved in interventions designed to change human behaviour and support the adoption of clinical programmes, leading to the publication of rich taxonomies and frameworks ^7, 9^. These frameworks have been used to map how different ingredients lead to mechanisms of action and to compare similar interventions to understand why some are more effective than others ^10– 12^. It has also become increasingly commonplace for the reporting of active ingredients involved in non-pharmacological clinical trials to be mandatory in journals. This has led to the development of the Template for Intervention Description and Replication (TIDieR) checklist which prompts the user to consider materials (physical or informational), procedures (activities or processes), people, and environment to describe an intervention ^13^, all of which could be considered ‘active ingredients’.

Whilst such work has proved valuable across multiple domains of health sciences, the linearity implicit in these kinds of implementation frameworks (i.e., that you can separate out components, describe them, and think of them as distinct from mechanisms and outcomes) has been argued to be theoretically reductive elsewhere, particularly in the social sciences. For example, linear frameworks are considered unable to articulate tacit social factors such as structural forces that shape interactions (e.g., Bourdieu’s field theory; 14) or the co-construction of meanings at an intersubjective or group level. In relation to the latter, Acord and DeNora ^15^ have argued that the meanings of arts encounters emerge within interactions. There is no simple cause and effect relationship whereby an art object causes an outcome, but rather it is the result of a ‘matrix of social relations and things’ where an object enables, as opposed to causes, forms of activity (p. 228). In this sense, any change (i.e., a health impact) that emerges is produced within a complex situation of engagement and meaning making ^16^. Similarly, Tan ^17^ drawing on Deleuze and Guattari’s concept of ‘assemblage’ has posited that experiences are afforded and affected by assemblages viewed as ‘encounters with a collection of animate and inanimate things’, theorising that there may be a ‘network of elements’ that prompt a range of experiences and opportunities for activity participants (p. 81). On this view, health and wellbeing outcomes are afforded by these elements and their relations ^17, 18^.

 However, implementation science does not sit at odds to these theorisations when combined with approaches from complexity science, incorporating an understanding of real-world, complex and dynamic systems (see 19, p. 7). Interventions that are considered ‘complex’ (such as an arts intervention) can be difficult to standardise in their design and delivery because of the wide range of *interconnected* components involved in prompting multiple mechanisms ^5, 6^. Indeed, as complexity science theorises, there may be multiple ingredients, mechanisms and multiple simultaneous causal strands required for an intervention to be effective ^20^, and ingredients and mechanisms may be indistinguishable for participating individuals due to their interdependence on another ^5^. Further, and in line with the social sciences, complexity science acknowledges emergent outcomes and relationality ^21^. For example, whilst identifying and articulating components is important, the agents and artefacts themselves may be considered ‘secondary to the relationships between these components’ ^21^. That is, they may combine or work together to coproduce health outcomes or create new phenomena as a result of their interactions.

 Applying a hybrid of factors simultaneously from both complexity science and implementation science involves methodological pluralism which has been argued to be compatible with pragmatism ^22^. This means a move away from linearity and the identification of active ingredients as objective components (as they may be considered within positivism) and focusing on applied research and multiple forms of knowledge creation ^22^. Taking this dual lens, the metaphor of ‘active ingredients’ can still be seen to have much to offer to our understanding of what *mix* of components (or interconnections between them) may be needed by arts activities to activate different mechanisms of action that can improve health and wellbeing, whilst acknowledging that this process is not simple or linear ^5^. Articulating ingredients can become not just a tick-box exercise but part of an in-depth exploration into the inner workings of an arts activity using a flexible and adaptive approach ^21^. Indeed, several studies have explored the identification of active ingredients in view of how they may need to combine together or work within a dynamic system to better understand how to optimise complex interventions. This includes interventions in the context of playgroups ^23^, paediatric rehabilitation programmes ^24^ and physical activity interventions ^11^.

### The active ingredients of arts activities

The endeavour to define how the qualities of arts activities lead to certain kinds of experiences in itself is not new. The definitions of what ‘art’ is and its characteristics can be traced back to Plato and have since been a continuing theme of philosophical debate ^25^. However, as work into the design and delivery of arts programmes to support health and wellbeing has proliferated, there have been increasing calls for more attention around identifying the ingredients of these programmes that help to achieve a health impact. Indeed, a recent review of culture on referral programmes (also known as arts on prescription or social prescribing programmes) noted that ‘there is a lack of exploration into the arts and cultural programmes themselves’ ^26^. A number of studies have explored the components of activities such as art therapies ^27^, including dance/movement therapy ^28^, and music therapy ^29^, the visual arts ^30^, and documentary media ^31^. Such work differs from that of defining ‘art’ more generally as it focuses on those ingredients considered relevant to achieving a health impact (i.e., those that are ‘active’) rather than all components of the arts activity. However, such work has generally described ingredients on a project-by-project basis, meaning they are only relevant for specific art forms. Whilst this can help develop appreciation of the nuances and specifics of each art form, it also means that the language used between art forms can vary, resulting in difficulties comparing findings from different studies and articulating if and how specific arts projects align or differ from one another. Recently, there have been attempts to draw some of the most common ingredients together ^2, 32^, and to group them into higher order themes such as ‘participants’, ‘environment’, and ‘quality of art activity’ that could apply across arts interventions more broadly ^17^. However, we still lack a comprehensive framework mapping all the potential active ingredients of arts in health programmes. 

Such a mapping exercise is needed to advance theory, research and practice. Theoretically, an integrative mapping exercise of potential active ingredients across disciplines encourages interdisciplinary learning, whereby knowledge of important components in one discipline may augment and explain those of another, thereby breaking down disciplinary barriers. Moreover, in view of the limited knowledge currently available, it seems likely that there are many active ingredients that are yet to be identified. From a research perspective, a comprehensive mapping could provide a unified approach to describing ingredients and allow the direct comparison of different arts interventions to identify where activities align or differ. This could, in turn, develop the understanding of how and why certain arts activities may activate certain causal mechanisms. From a practice perspective, mapping ingredients can support in the design, implementation, and evaluation of arts interventions; for example, by helping practitioners to consider which ingredients to include within an intervention to achieve particular health outcomes or to assess how closely the delivery of an intervention matches original plans.

Considering the evidence above we therefore set out to 1) carry out the most comprehensive mapping of ingredients in arts in health activities to date, developing them into a new theoretical framework (the INNATE Framework), and 2) cocreate a usable toolkit to support with the design, implementation, and evaluation of arts in health activities.

## Methods

Drawing on the principles of implementation science and complexity science, we aimed to synthesise active ingredients in the literature across art forms with new empirical data, carrying out a collaborative and comprehensive mapping exercise to develop a new framework. Rapid Appraisal was adopted as a methodological approach because it is not tied to a particular epistemology, is team-based, and is iterative, whereby active ingredients can be identified and developed throughout the analytic process ^33^. We engaged in a three-phase process of: 1) a scoping review to inform the development of an initial framework; 2) a consultation on the initial framework; and 3) analysis of consultation responses and construction of the INNATE framework.

Across these phases, we focused specifically on ‘arts in health’ activities. Here, our definition of ‘arts’ includes activities involving participation in arts and other creative activities, as well as engagement with culture and heritage. ^2^ Our definition of ‘arts in health’ was interventions that are designed and delivered either in healthcare contexts (e.g., a music programme delivered in a peri-operative setting), as part of healthcare referral schemes (e.g., a referral to a community choir by a healthcare professional), or with specific health or wellbeing outcomes in mind (e.g., an online dance intervention for chronic pain). ^3^

### Phase one: Scoping review

Our research began with a scoping stage to ‘map and categorise’ relevant literature to examine the ‘landscape’ of our area of study ^35, 36^, cataloguing ingredients already identified and laying the groundwork for phase two. We drew on methods identified by Arksey and O’Malley ^35^ to:

1. *Identify our research question* which we formulated as ‘what are the active ingredients of arts in health activities?’
2. *Identify the relevant literature.* We created a table of key word searches (see Additional File 1, table 1.1 ^37^) and used these to search databases including Google Scholar, PubMed, ScienceDirect, Web of Science, Scopus, and UCL Explore. We included grey literature and sought to include a range of methodologies and populations.
3. *Chart the data.* We read through the literature sourced and extracted any findings which we identified as key active ingredients in view of our definition of this term, as outlined in the introduction. We then listed the active ingredients that had been identified in an excel spreadsheet, noting the author(s) of the research, the arts activity studied, and the key ingredients identified (see Additional File 1; table 1.1 ^37^).
4. *Collate and summarise.* Through mind mapping using the software Keynote, we categorised the ingredients we had identified into three emergent categories: project, people, and contexts. Next, we used these headings to reorganise the active ingredients in our spreadsheet for use in the next phase of the project. As many of the studies were framed within different disciplinary contexts and/or employed theory to conceptualise ingredients, DF (PhD, an Associate Professor with a background in psychobiology and epidemiology) and KW (PhD, a Research Fellow with a background in qualitative social science) discussed and refined the language and concepts together as a first step to embedding these ingredients within the context of implementation science. We also added definitions and examples next to each of the active ingredients to ensure clarity of expression.

### Phase two: Consultation

The initial mapping spreadsheet from phase one was developed into a more accessible worksheet (see Additional File 2 ^37^) and shared with 64 participants prior to them taking part in one of 10 semi-structured focus groups. The focus groups lasted 90 minutes each, were conducted between December 2020 and March 2021 online using Microsoft Teams, and were facilitated by one female researcher (KW). The number of participants ranged from 5 to 8 per group. Participants included people who design and/or deliver arts and cultural activities who were able to consider how their activities may contribute to health and wellbeing outcomes, as well as those involved in relevant research or arts programme management (See Table 1 for participant details, with further information in Additional File 3; table 3.1 ^37^). Participants were approached by email and recruited via the MARCH Research Network, one of eight national mental health research networks funded by UK Research and Innovation (UKRI) focused on social, cultural and community engagement, and through the professional networks of the research team. Recruitment began in December 2020 and continued through to the end of the data collection period at the beginning of March 2021. Participants were provided with an information pack ahead of attending a focus group which included: an explanation of the rationale for the research, a brief outline of what implementation science is, a logic model linking the arts to health outcomes (building on the model presented in the 2019 World Health Organisation (WHO) Health Evidence Network Synthesis Report ^2^), and the questions that would be asked in the focus group. They were also informed that the groups would be led by KW. During each focus group, the worksheet was used as a prompt for questioning, discussing the suitability of the language used for each active ingredient, as well as identifying any missing ingredients. Extensive notes of the discussion were taken by the facilitator (KW) and transcripts were generated automatically by Microsoft Teams. All focus groups were audio recorded. The topic guide is included in Additional File 3; 3.2 ^37^.

**Table 1.** Job role and arts/cultural activities engaged in of focus group participants.

Number of participants	64
**Job role**	
Artist/cultural practitioner	37
Arts/cultural manager or administrator	14
Researcher/Academic	8
Healthcare professional	2
Other	3
**Where arts/cultural activity normally** takes place *	
Community setting	50
Public health setting	26
Arts/cultural venue	35
Education setting	31
Online/digital setting	41
Criminal justice setting	1
Religious setting	1
Other health or care setting	2
Other	4

*NB. Participants could select all that applied for this question

### Phase three: Analysis and construction of the INNATE Framework

**Figure 1.**
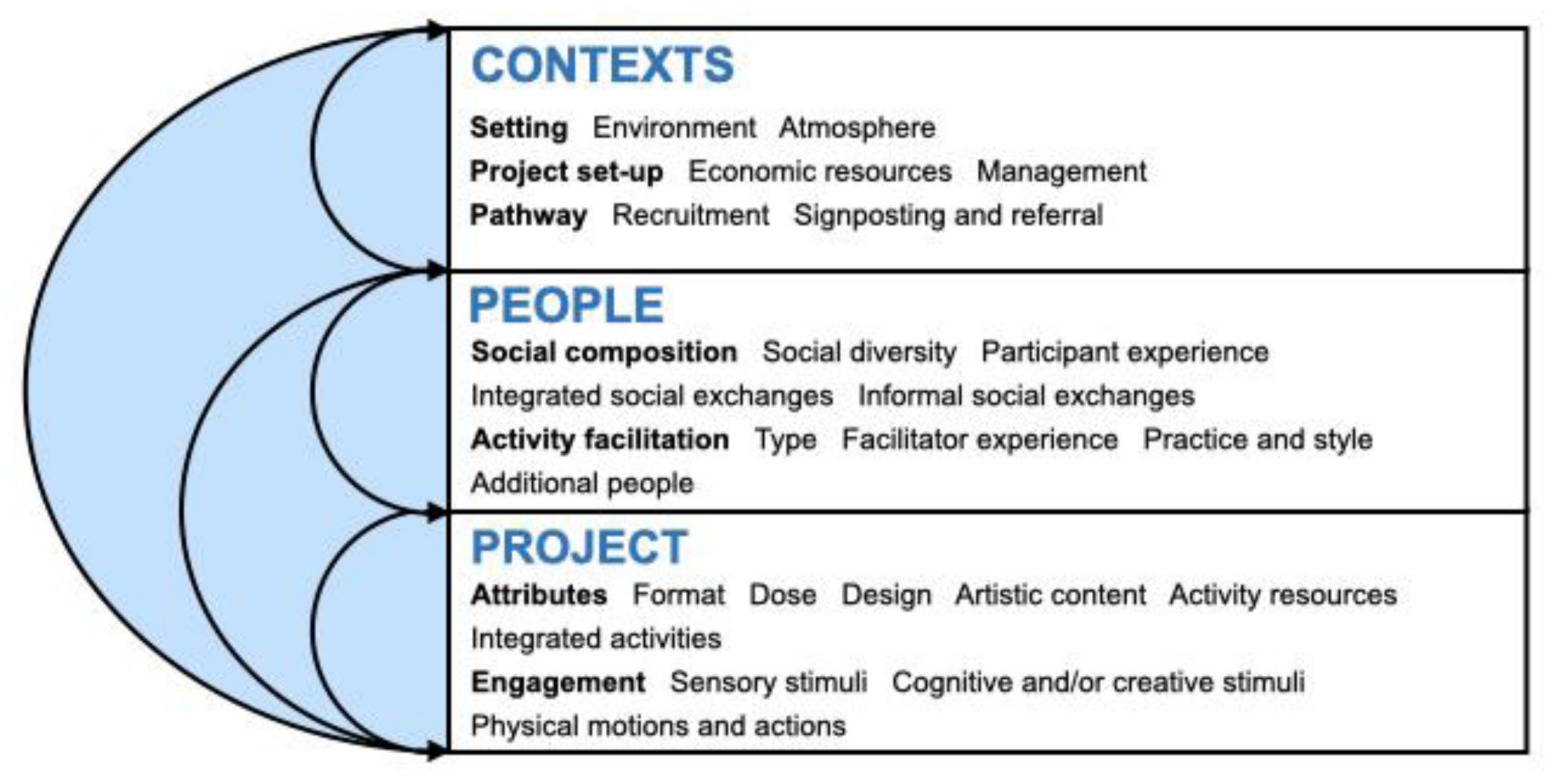
The INNATE Framework: active ingredient categories and subcategories, showing interconnections and feedback loops.

Data collection (phase two) and analysis were carried out in parallel over an intensive 12-week period, with the worksheet updated after each data collection point, until we had reached saturation by the final focus group where no new ingredients or substantial changes to existing ingredients were identified. To do this, RAP sheets (Rapid Assessment Procedures; see example in Additional File 3; table 3.3) were used to systematically document and synthesise emergent findings throughout the study ^41, 42^. The RAP sheet consisted of a table with thematic headings of interest, whereby summaries of findings were inserted into the columns by the facilitator (KW) after each focus group, with the summaries created through triangulation of reading researcher notes and listening and reading relevant sections of focus group audio and rough transcripts respectively. Every week, the core research team (DF & KW) met to discuss the emergent findings and to review and modify the worksheet as needed based on the empirical data, alongside updating the mapping Excel spreadsheet. Where there were disagreements over particular ingredients in the focus groups, we resolved issues through a combination of revisiting the literature and discussions both within and across different focus groups and team meetings until consensus was reached. At the end of the focus groups, we developed a visual model ( Figure 1) and all identified ingredients were refined through final input from the co-investigators of the MARCH Network and feedback from focus group participants.

## Ethical statement

The study was approved by the UCL Research Ethics Committee (Project ID: 19105/001), and all focus group participants provided written informed consent. Participants were made aware that the research aimed to identify and explore the different components that make up arts and cultural activities, and that their personal information would remain confidential. They were also informed that their focus group responses would be anonymised. Participants consented to the results of the study being submitted for publication in peer-reviewed academic journals and were provided with an option to receive the final report after publication.

## Results

We identified and refined 139 active ingredients of arts in health activities that may prompt mechanisms of action that affect health and wellbeing across three thematic categories: project, people, and contexts. We also developed a visual model (see Figure 1) to show how ingredients may overlap, interconnect, and feed into one another. The model and summary of the active ingredients’ categories are provided below, with the full lists of ingredients with definitions included in Additional File 4 ^37^.

### Project

Active ingredients in the project category relate to the *attributes* (qualities and characteristics) of an arts or cultural activity, as well as the kinds of stimuli involved in prompting *engagement* with that activity. These are the components that relate directly to the content of the activity itself, intrinsic to describing what the activity is ( Table 2).

**Table 2.** Categories and subcategories of active ingredients in the ‘project’ category.

CATEGORIES	SUBCATEGORIES
Attributes *Qualities and characteristics of the activity*	
**Format** *Relating to the arrangement, style and type of activity*	**Mode**
	**Synchroneity**
	**Activity level**
**Dose** *The amount of activity(ies) received by participants*	**Frequency**
	**Duration**
	**Maintenance**
**Design** *Relating to a structural plan for the activity which* *may or may not be adaptable*	**Structure**
	**Guiding**
	**Project approaches**
	**Personalisation**
	**Challenge**
	**Goal orientation**
	**Feedback**
**Artistic content** *Relating to the artistic dimensions of the activity*	**Genre**
	**Multi-modality**
	**Activity type**
	**Themes**
**Activity resources** *Physical, conceptual or informational materials used/* *employed in the delivery of the activity*	**Activity consumables**
	**Props**
	**Products**
	**Performances**
**Integrated activities** *Activities that are integrated within the arts/cultural* *activity(ies)*	**Psychosocial support**
	**Allied therapies**
	**Health education**
	**Spiritual or holistic practice**
	**Socially-engaged practice**
Engagement *Stimuli prompting active involvement in the activity*	
**Sensory stimuli** *Objects, actions, materials or experiences that* *activate the senses as part of the activity*	**Vision (sight)**
	**Auditory (hearing)**
	**Olfactory (smell)**
	**Gustatory (taste)**
	**Tactile (touch)**
**Cognitive and/or creative stimuli** *Objects, actions, materials or experiences that* *activate cognitive processes as part of the activity*	**Involvement of the imagination**
	**Emotional stimuli**
	**Cognitive stimuli**
	**Aesthetic engagement**
	**Pleasure**
	**Participant choice**
**Physical motions and actions** *Physical, bodily motions or actions employed as part* *of the activity*	**Proprioception (kinaesthesia)**
	**Movement**
	**Physical exercises**

The *attributes* encompass both logistical and artistic characteristics. The former includes the *format* of the activity (such as whether it is an in-person or virtual engagement), the *dose* of the activity received (relating to how much and how often participants are exposed to the activity), and the *design* (or structural plan) of the activity (which may or may not be adaptable). The latter includes the *artistic content* (relating to the genre, type of activity, and themes or subjects drawn upon), as well as the *activity resources* used or employed (whether physical, conceptual, or informational). In addition, the attributes relate to any *integrated activities* (non-arts programmes or experiences) that form part of the project, including psychosocial support such as Cognitive Behavioural Therapy (CBT) or meditation, allied therapies such as physiotherapy or occupational therapy, health education programmes, or spiritual or holistic practices.

*Engagement* denotes active ingredients relating to stimuli prompted by the activity. This includes *sensory stimuli* (vision, auditory, olfactory, gustatory, and tactile), *cognitive or creative stimuli* (e.g., mental processes, images and pleasurable experiences), and *physical bodily motions or actions* that are integrated into their content (e.g., awareness of the body, movements or physical exercises).

### People

Active ingredients in the people category denote *social composition*, relating to how people interact through engagement with the activity and who is involved in this interaction, as well as the *activity facilitation*, concerning the people who lead, guide, or facilitate the participant-facing aspects of the activity ( Table 3).

**Table 3.** Categories and subcategories of active ingredients in the ‘people’ category.

CATEGORIES	SUBCATEGORIES
**Social composition** *Relating to how people interact through engagement with the activity and who is involved in* *this interacting*		
**Social diversity** *The people involved in the activity*	**Presence of others**
	**Shared attributes**
	**Distinct attributes**
	**Personal attributes**
**Participant experience**	**Activity experience**
	**Health experience**
	**Lived experience**
	**Relationship to others**
**Integrated social exchanges** *Social exchanges (face to face or digital) between * *participants that are part of or integrated into the* *activity*	**Shared focus**
	**Shared activity**
	**Social exchanges**
	**Structured social time during activity**
	**Structured social time outside activity**
	**Communications**
**Informal social exchanges** *Social exchanges (face to face or digital) between* *participants that are not planned as part of the activity*	**Unstructured social time during activity**
	**Unstructured social time outside activity**
Activity facilitation *Relating to the people who lead or guide the outward facing aspects of the activity (i.e. not the* *administrative aspects but the activity facilitation)*	
**Type** *Relating to the kind of leadership employed to deliver* *the activity*	**Facilitator(s)**
	**Co-production**
	**Number**
	**Professionalisation**
	**Training**
	**Consistency**
**Facilitator experience** *Approaches and/or experiences that a facilitator may* *bring to an activity*	**Activity experience**
	**Health experience**
	**Lived experiences**
	**Relationship to others**
**Practice and style** *The manner in which the activity is delivered*	**Technique**
	**Personal attributes**
	**Values-directed focus**
	**Outcomes-directed focus**
	**Person-centred focus**
	**Autonomy-directed**
	**Equality, diversity and inclusion**
	**Safety**
	**Tailoring**
**Additional people** *Staff or other people that support or co-lead the activity*	**Presence of volunteers**
	**Presence of healthcare professionals**
	**Presence of others**

 The *social composition* of an activity refers to the people involved in an activity and components of the activity that involve social interaction. This is characterised by *social diversity* (who is present and their attributes), and *participant experience* (whether those involved have previous experience of engaging with the activity, health conditions or contexts, their own lived experience, or already know others engaging). Social exchanges within this category can be part of or *integrated* into the activity (social interactions that are dimensions of the activity or structured social time) or *informal* (unstructured social time such as refreshment breaks).

*Activity facilitation* focuses specifically on people who are ‘in the room’ (whether in-person or digitally) actively delivering the activity. This overarching category is broken down into four sections. *Type* denotes if an activity involves facilitation and, if so, what form this takes (e.g., who and how many people facilitate, lead, or guide an activity and the consistency of this facilitation). *Facilitator experience* relates to the approaches and experiences that a facilitator may bring to an activity (e.g., prior experience leading similar activities, working with specific populations, lived experience, previous relationships to activity participants, and specific traits that affect how they approach facilitation). One’s experience may also intersect with the *practice and style* in which the activity is delivered (such as whether facilitator(s) use particular techniques or have personal attributes that affect how they facilitate or express foci relating to values, outcomes, tailoring, equality, or participant preferences and safety). The *additional people* of an activity may support, co-lead, or just be present at an activity (e.g., volunteers, healthcare professionals or observers).

### Contexts

Active ingredients in the contexts category relate to the activity *setting* comprising the aggregate of place(s), things, surroundings and feelings that make up the situation and *project set-up,* such as the structure, processes and/or systems which surround the delivery of the activity ( Table 4).

**Table 4.** Categories and subcategories of active ingredients in the ‘contexts’ category.

CATEGORIES	SUBCATEGORIES
**Setting** *The aggregate of place(s), things, surroundings and feelings that make up the* *situation of the activity*	
**Environment** *The circumstances, objects, and conditions which* *make up the surroundings of the activity*	**Location**
	**Basic features**
	**Attractiveness**
	**Situation**
	**Time and day**
	**Access**
	**Privacy**
**Atmosphere** *The character, feeling, or mood of a place or* *situation where the activity takes place*	**Comfort**
	**Belonging**
	**Familiarity**
	**Ambiance**
	**Organisation**
**Project set-up** *The structure, processes and/or systems which surround the outward facing delivery* *of the activity*	
**Economic resources** *Relating to economic resources connected to the* *activity and its delivery*	**Participant charges**
	**Project funding**
	**Fees**
	**Longevity**
	**Environmental sustainability**
**Management** *Relating to the person, people, group(s) or* *company(ies) in charge of organising the activity*	**People**
	**Affiliation**
	**Branding**
	**Collaboration**
	**Patient and Public Involvement**
**Pathway** *Relating to the partcipant(s') route into or out of the activity*	
**Recruitment** *How participants find out about or are enrolled* *into the activity*	**Formal referral**
	**Informal referral**
	**Choice**
	**Advertising**
**Signposting and referral** *Signposting to services, resources, support, or* *advice beyond the activity itself*	**Inter-sector signposting**
	**Health-sector signposting**
	**Social signposting**
	**Other-sector signposting**
	**Safeguarding referral**

 The setting of the activity is characterised by the activity *environment* (i.e., the location, access and functional and aesthetic components) and *atmosphere* (the character, feel, or mood of the place or situation of the activity). The project set-up includes both the management of the activity itself, as well as the unique pathways into and out of the activity that participants may follow. This comprises *economic resources* (charges for participation, project funding, and project resources) and project *management* (such as affiliation, branding, collaboration and patient and public involvement). Related to this, *recruitment* (including formal and informal referrals) and *signposting and referral* (e.g., to other arts activities, to medical or psychosocial services, or to welfare or caring support) denote the pathways into or out of the activity.

## Discussion

This study embarked upon an empirically grounded mapping exercise to identify the active ingredients of arts in health activities. Through this process, the study identified 139 potential active ingredients within the overarching categories of *project, people*, and *contexts*, presented as a framework for design, implementation, and evaluation to support those delivering arts and cultural activities.

### Comparison to previous research

Some of the ingredients presented here were identified in our scoping review (see Additional File 1; table 1.2), which we refined through phases two and three of our study. As we aimed to provide language that would be relevant to a range of arts in health activities, in some cases, this meant translating context-specific or theory-laden language into general terms (e.g., ‘therapist’ into ‘facilitator’; ‘daily session’ into ‘dose’), and aggregating or excluding ingredients through our iterative focus group procedure, based on the consensus of our participants. The worksheet created through this study (see Additional File 2) also provides a similar reporting format to the TIDieR framework ^13^, but focuses specifically on ingredients relevant to arts in health, enabling greater nuance. Our framework is a substantial advance on previous conceptualisations and toolkits because it comprehensively maps ingredients relevant to all arts in health activities, providing bespoke prompts in a worksheet for use in this field.

Some active ingredients presented in this framework have also been conceptualised in relation to other psychosocial interventions for health. For example, a scoping review of therapeutic playgroups for children identified peer support and facilitator training as important to a family-centred approach, echoing ingredients within the ‘social composition’ and ‘leadership’ sections presented in this framework ^23^. Ongoing research funded by the Wellcome Trust has identified safety, trust, positive connection, and co-designed spaces as active ingredients for supporting neighbourhood connections for young people’s mental health, echoing ingredients in our people and contexts categories ^43^. Further, a meta-analysis of interventions employed to increase physical activity amongst aging adults identified 20 intervention components, including commitment, classes at set times, feedback on performance, goal setting, health education information, and social support ^44^. Whilst the language here is tailored to physical activity, some dimensions of these components can be viewed as similar to our identified ingredients of dose, feedback, goal orientation, integrated activities (e.g., health education) and social exchanges. These are just a few examples but serve to highlight that there may be some active ingredients of arts and culture present in other interventions.

However, our research highlights certain ingredients that may be unique to arts and culture. The notion of there being something distinctive about arts activities has been proposed before. Arts engagement has been described as having an ethereal or indescribable quality, such as in the context of group singing interventions which have been viewed as ineffable ^45^, with the arts intervention itself considered a ‘complicated situation’ ^16^. Whilst recognising that articulating what is ‘in’ arts and culture is notoriously difficult, we have captured some of these core ‘artistic’ ingredients across a range of components that may combine together to explain differences between the arts and other psychosocial activities. For example, artistic content (which can be multi-modal), activity resources, sensory stimuli, cognitive stimuli, stimuli prompting aesthetic engagement or pleasurable experiences, facilitators drawing upon artistic practice, and the attractiveness of the environment were all identified. Whilst some of these may be present individually in other interventions, the essence of the ‘artistic’ experience may also emerge via the specific combination of these and other ingredients provided through an arts activity, such as individuals engaging in cognitive processes such as problem solving within the context of multisensory engagement ^46^. In this sense, the simultaneous presence of many of these ‘artistic’ ingredients and their interactions with one another present a way of differentiating the arts from other interventions (e.g., from psychological interventions such as counselling or from social activities such as group sports).

### Implications for future research

The ability to specify ingredients according to a detailed framework should aid in the comparison of different arts in health activities; for example, comparing two similar interventions (e.g., an experimental group taking part in a dance for Parkinson’s programme within a randomised controlled trial and a control group taking part in an exercise for Parkinson’s programme). Such comparisons could support more detailed reporting within process evaluations, aiding the interpretation of study findings. In other instances, the framework may help in identifying changes to an intervention as a study evolves. As an example, we have demonstrated how the worksheet could be used to compare the active ingredients of an in-person activity with a version adapted for an online format in the Additional File 5 ^37^. In multi-site studies that involve the delivery of the same intervention in different locations, the framework could be used to tailor interventions and highlight adaptations that are essential to meet local needs. Identifying ingredients after a multi-site study has already been delivered may also highlight changes to ingredients that happened organically, bringing to the fore reasons why it might be difficult to combine findings from across sites. Such specificity in reporting could improve the replication of studies.

Second, the specification of precise active ingredients may facilitate research exploring how ingredients activate specific causal mechanisms linked to health outcomes. This is an important step in being able to design activities that aim to modulate specific mechanisms and outcomes. Nonetheless, it should be remembered that arts activities are complex interventions. Whilst active ingredients have been presented in our tables as separate components, these ingredients may overlap or feed into one another, working together to prompt causal mechanisms. Our research identifies a large range of active ingredients that may need to work together to influence health outcomes. The combination of these ingredients may lead to more than the sum of the individual parts ^47^. Thus, studies that attempt to manipulate specific ingredients in isolation from one another may risk altering other ingredients in an activity ^48^ and may not be able to reliably manipulate specific mechanisms ^20^. Research exploring the interconnection between ingredients and mechanisms needs to take a pluralistic lens, considering how the removal or substitution of one ingredient affects other ingredients, and look more broadly at the effect of this on multiple mechanisms and outcomes to notice unanticipated changes.

Additionally, active ingredients may vary not just at an activity level but also for individuals taking part in an activity. For example, pre-existing friendships between activity participants (the active ingredient of ‘relationship to others’) may only be present for some participants. The effects of such ingredients on causal mechanisms may be affected (moderated) by the broader context. Whilst some of these factors are captured within the ‘contexts’ theme, we cannot view arts in health activities as existing in isolation from the broader macro environment ^49^. Individuals’ participation in the arts is affected by their own life histories and wider societal historical, political, economic, temporal and spatial factors ^50^. Complex interventions are recognised as existing in a state of equilibrium rather than stasis ^51^. So ingredients that have the ability in the current context to activate specific causal mechanisms may not continue to have such potency as contexts evolve ^52^. For example, the psychological impact of engaging in digital arts activities when they were novel technologically is likely very different to the same experience now when such activities are ubiquitous. Research needs to acknowledge these wider factors.

Finally, this framework was developed with a specific focus on the active ingredients of arts in health interventions. However, it may also have a relevance to research seeking to understand how more ubiquitous engagement in arts and culture as part of daily life (not for specific health purposes) can nonetheless still lead to health and wellbeing outcomes. Future work is encouraged that explores the validity of applying this framework to such contexts.

### Implications for practice

The INNATE framework may support in the design and evaluation of arts in health interventions with a specific target health outcome. By presenting a clear way of thinking about ingredients, it could be used to provoke discussions on how an activity should be established and run to be optimised for different participant groups. For example, it could aid project planning and practice-based evaluation by helping practitioners identify intervention inputs and mechanisms. Further, given that there are well-recognised differences in language between and across art forms, disciplines, and sectors (e.g., arts, health and social care), the framework may help to establish common terminology between multi-disciplinary teams as interventions are designed. However, users should be cautioned against feeling that every ingredient needs to be present in an activity. The presence of more ingredients does not necessarily imply stronger effects on health. Additionally, the specific combination of ingredients is likely as important as the presence or absence of individual ingredients.

Second, engaging in identifying ingredients for an existing intervention can prompt processes of reflexivity. Reflexivity denotes ongoing self-reflective processes on one’s research or practice as a form of ‘critical self-awareness’ ^53, 54^. This process is becoming increasingly employed by arts practitioners as a mode of reflective enquiry ^53, 54^, drawing on its longer history of use within arts therapies ^55^. Many of our focus group participants reported how the prompts in the worksheet facilitated these kinds of reflexive processes, whereby contemplating which ingredients were present in their activities enabled reflection on what works and why, thereby improving activity delivery and evaluation.

## Limitations

This study has a number of strengths; it presents a new theoretical framework for identifying the active ingredients of arts in health, constructs a shared language for identifying active ingredients, and has developed a worksheet that can be used in practice, all of which can support in advancing the design, delivery, and evaluation of arts in health interventions. However, it also has several limitations.

Firstly, although we used a wide range of search terms in our literature search, the diversity of language currently used to explore ‘ingredients’ means we may have missed specific papers. Similarly, our focus groups involved people from a broad range of artforms, but arts and cultural engagement is diverse. Therefore, it is possible that certain ingredients were omitted from our mapping exercise. Additionally, we focused on the ingredients as viewed from the perspectives of those designing and delivering arts in health interventions rather than those participating. Much like in cooking, the ingredients as added by the chef will be different to those tasted by the diner; ingredients within arts and cultural interventions are ‘dynamic systems’ and not ‘static states’ and are modified by their presence alongside one another, the context in which they are presented and the experiences of the individual engaging ^3, 48^. Extending this culinary metaphor, we must be cautious about oversimplifying the concept of active ingredients to a ‘recipe’: this framework focuses on key ingredients but it is not an exhaustive list. Specific nuances in the design and delivery of an activity and subtle shifts in the processes of engagement and the pathways that individuals take into and out of engaging over time are often critical to the way an arts activity is experienced by and impacts on individuals. However, from an implementation perspective - the lens from which this study was designed and conducted - being able to articulate the larger ingredients presented here is a major step forwards in how we discuss arts in health interventions and will be critical to the work seeking to expand and sustain arts in health programmes. INNATE is presented here as a framework that will expand and evolve over time.

 Second, the inclusion of an ingredient in this framework does not imply that it has the potential to lead to health outcomes. We focused on ingredients common in arts in health activities but, due to limitations of existing research, it is not currently clear whether all of these have the potential to influence causal mechanisms or health outcomes. Some may be vital ingredients for specific outcomes, whilst others may play a role in facilitating specific mechanisms or provide important context within which the vital ingredients can be experienced, and others may have little or no effect. Our research does not recommend one specific ingredient over another, rather outlining the range of ingredients to be considered when designing and implementing arts in health activities. Future research is encouraged to identify which ingredients and their combinations are most important for specific mechanisms and outcomes.

 Third, within different disciplinary silos and sectors, the language used for certain ingredients will sit more comfortably than for others. We recognise that the concept of ‘active ingredients’ is a metaphorical device that is used within particular healthcare contexts and may be viewed as too narrow by those implementing arts and cultural activities across other disciplines and sectors. However, our framework is not intended to replace vocabularies unique to different art forms or sectors, nor will every active ingredient be relevant to every kind of intervention. Rather, in view of the wide range of people from different sectors who work in the field of arts in health and the diversity of activities delivered, it is hoped that it can be used to support dialogue across domains, acting as a ‘common dictionary’.

 Finally, in this paper, we have separated out active ingredients from mechanisms of action ^3^. This could falsely give the impression that in the real world, ingredients exist separately from the mechanisms they activate in a simple, linear model. However, the concept of 'active ingredients’ is a theoretical tool to support with implementation, rather than markers of an objective reality, and the boundaries between some of our active ingredients and mechanisms may be viewed differently within other disciplines. The INNATE framework presented here is designed to be used in conjunction with an understanding of how ingredients activate mechanisms of action that lead to health outcomes. Specifically, the Multi-level Leisure Mechanisms Framework ^3^ proposes a model for how active ingredients in arts interventions can affect health outcomes via different categories of mechanisms of action, all situated within a complexity science framework that considers micro-, meso-, and macro-level moderators. We encourage that the INNATE and Multi-level Leisure Mechanisms Frameworks are read and applied together.

## Conclusion

This study has identified 139 active ingredients of arts in health activities, mapping out a comprehensive list of interconnected ingredients, alongside constructing a worksheet to support in the replication, evaluation, and scaling-up of interventions. Whilst recognising that no framework can capture every ingredient of a complex activity such as arts engagement and that more research is needed to explore the connections between ingredients, mechanisms and outcomes, our mapping exercise is the most extensive to date. It is hoped that our research will lay the foundation to build upon our theoretical knowledge of what it is about arts and cultural activities that enables them to affect our health.

## Data availability

The raw data from this study is not being publicly archived for use by other researchers because the data contains information that could compromise the privacy of research participants. The UCL Research Ethics committee have restricted the use of the data to UCL researchers only. To discuss the conditions of this availability further, please email k.warran@ucl.ac.uk. 

### Extended data

OSF: ‘Supplemental materials for paper: What are the active ingredients of ‘arts in health’ activities? Development of the INgredients iN ArTs in hEalth (INNATE) Framework’. DOI 10.17605/OSF.IO/Z3QRE ^37^.

This project contains the following extended data:

- Additional file 1 – Scoping review.docx (Scoping review additional information including key word searches and results of the initial scoping review)
- Additional file 2 – Worksheet.docx (Worksheet to identify the active ingredients of arts for health activities)
- Additional file 3 – Focus groups.docx (Focus group additional information including additional details about participants, the topic guide used and an example of a RAP Sheet)
- Additional file 4 – Active ingredients of arts in health.docx (Full table of active ingredients identified including descriptions)
- Additional file 5 – Application of the worksheet.docx (Example application of the active ingredients worksheet to show how it can be used to compare the active ingredients of two interventions, including colour chart comparison)

Data are available under the terms of the Creative Commons Attribution 4.0 International license (CC-BY 4.0).

## Authors' contributions

DF conceived the idea for the study, with KW and AB collaborating on its design. KW led on the empirical data collection and analysis, with weekly input at team meetings with DF. KW drafted the report with input from DF and AB.

## Footnotes

^1^ Similar to the roots of ‘active ingredients’, the term ‘mechanisms of action’ also stems from pharmacological research to describe how a drug exerts an effect – it’s underlying processes. However, its use has become increasingly widespread within the study of complex interventions to understand how these interventions affect health. This has recently included the study of the mechanisms by which leisure activities (which includes arts activities) affect health framed within complexity science, leading to the development of the Multi-level Leisure Mechanisms Framework ^3^. This framework informed our understanding of ‘mechanisms of action’ adopted for this study.

^2^ The definition of ‘art’ is subject to ongoing debates and cross-disciplinary conflicts ^34^. However, despite conceptual complexities, a number of cross-cultural characteristics have been recognized as fundamental to art, such as the presence of an art object which provides meaningful, imaginative, and emotional experiences ^2^. For this study, we defined art in terms of the *kinds of activities* that provide these experiences (as opposed to conceptual or abstract concepts of ‘art’). In relation to health research, these activities include: i) performing arts activities such as singing, dancing and acting; ii) visual arts participation including drawing, painting and crafts; iii) digital arts activities including photography, animation and film-making; iv) literary arts such as reading and creative writing; v) cultural engagement such as going to museums, galleries and concerts; and vi) heritage engagement, such as visiting monuments and stately homes ^2^. Within these categories there are a diverse range of different types of arts engagement that transcend cultural boundaries, covering both active engagement with the arts, such as participatory arts programmes, and receptive engagement, such as audiences viewing art objects ^2^.

^3^ There is no universal definition of ‘arts in health’ and there are a number of related concepts that are often used in different contexts to designate similar activities, such as arts for health, arts and health, and arts therapies, to name a few examples. We have specifically chosen to use arts *in* health to denote ‘the use of the arts as a way of supporting individual health or healthcare systems’ ^38^. This is in contrast to ‘arts and health’ which may be used more broadly to indicate any bringing together of the arts with health, going beyond a focus on health outcomes, or ‘arts for health’ which has connotations of advocacy ^38^. Our definition also includes arts therapies within it, viewing these activities as aligned with the aim of drawing on the arts to support health through the production of art work in healthcare ^39^. Further, we consider ‘health’ to encompass health prevention, promotion, management, and treatment, drawing on the World Health Organisation’s understanding of health as ‘a state of complete physical, mental and social well-being and not merely the absence of disease or infirmity’ ^40^.
