# Supplementary material for: What are the active ingredients of ‘arts in health’ activities? Development of the INgredients iN ArTs in hEalth (INNATE) Framework
Source: Wellcome Open Res. 2022 Apr 29;7:10. Originally published 2022 Jan 11. [Version 2] doi: 10.12688/wellcomeopenres.17414.2 (PMC9106992; doi:10.12688/wellcomeopenres.17414.2)
Supplement: Peer review history

# Peer review: Reviewer response for version 2

**DOI:** 10.21956/wellcomeopenres.19766.r50266

**Published:** 2022-05-13

**Authors:** Gunter Kreutz

**Affiliations:** 1 Department of Music, Speech and Music Lab, Carl von Ossietzky University Oldenburg, Oldenburg, Germany

I am satisfied with the changes and amendments. I see little scope of improving the manuscript in the current state and congratulate the authors to an important contribution to the field, in my view.

Is the work clearly and accurately presented and does it cite the current literature?

Yes

If applicable, is the statistical analysis and its interpretation appropriate?

Not applicable

Are all the source data underlying the results available to ensure full reproducibility?

Partly

Is the study design appropriate and is the work technically sound?

Yes

Are the conclusions drawn adequately supported by the results?

Partly

Are sufficient details of methods and analysis provided to allow replication by others?

Yes

Reviewer Expertise:

music psychology, music and health

I confirm that I have read this submission and believe that I have an appropriate level of expertise to confirm that it is of an acceptable scientific standard.

# Peer review: Reviewer response for version 2

**DOI:** 10.21956/wellcomeopenres.19766.r50265

**Published:** 2022-05-03

**Authors:** Melissa Forbes

**Affiliations:** 1 Centre for Heritage and Culture and School of Creative Arts, University of Southern Queensland, Toowoomba, Qld, Australia

The article is strengthened by the revisions, and I have no further comments.

Is the work clearly and accurately presented and does it cite the current literature?

Yes

If applicable, is the statistical analysis and its interpretation appropriate?

Not applicable

Are all the source data underlying the results available to ensure full reproducibility?

Yes

Is the study design appropriate and is the work technically sound?

Yes

Are the conclusions drawn adequately supported by the results?

Yes

Are sufficient details of methods and analysis provided to allow replication by others?

Yes

Reviewer Expertise:

practitioner-researcher in singing for health and wellbeing

I confirm that I have read this submission and believe that I have an appropriate level of expertise to confirm that it is of an acceptable scientific standard.

# Peer review: Reviewer response for version 1

**DOI:** 10.21956/wellcomeopenres.19253.r47984

**Published:** 2022-02-10

**Authors:** Melissa Forbes

**Affiliations:** 1 Centre for Heritage and Culture and School of Creative Arts, University of Southern Queensland, Toowoomba, Qld, Australia

This article presents the INNATE framework and toolkit for use in the design, implementation, and evaluation of arts in health activities. The framework is an important contribution to the field of the arts in health, as it synthesises into three categories the "active ingredients" of arts activities that lead to health and wellbeing effects - *project, people, *and *contexts*. These categories are useful, empirically-grounded thinking and design tools for arts in health practitioners, researchers and evaluators. They also stand as entry points for approaching and understanding the complex phenomena of arts in health activities.

 I suggest including a brief description of the research participants in the abstract (e.g. "64 arts in health researchers, practitioners and managers" or similar) so it is clear to the reader from the outset who the participants were.

 The background situates the contribution of the framework by explaining the use of the term “active ingredients” which has its basis in pharmacological research. The authors acknowledge, however, that within the social sciences, a linear concept such as “active ingredients” may not easily transfer to non-pharmacological interventions such as arts activities for health and well being which are complex systems.

 The authors might consider referencing the work of Camlin *et al. *(2020) ^1^ (either in the second to last or last paragraph of this section), which positions arts activities (and group singing in particular) as holistic and complex adaptive systems.

 The authors conclude this section by outlining how a hybrid conceptualisation of “active ingredients”—acknowledging how these are understood in both complexity and implementation sciences—provides a useful lens to understand the mix of components that activate the mechanisms which improve health and wellbeing.

 The methods and results are clearly articulated and reproducible with extended (but not raw) data included.

 Figure 1 - the INNATE framework - will be of particular use in the monitoring and evaluation of arts in health programs, as it can inform the development of an initial program logic/theory of change as well as provide a conceptual frame for program evaluation upon program maturity.

 The implications of the research for practice and future research, and limitations, are acknowledged and discussed thoroughly.

 Reading as a practitioner-researcher, the framework's categories and sub-categories ring true and appear comprehensive. One limitation of the framework could be that it does not necessarily identify a hierarchy of ingredients. For example, might facilitator experience in the "People" category play a more fundamental role in the success of an activity than say, the environment in the "Contexts" category? Or, for argument's sake, *vice versa*? The authors do however emphasize in the section on implications for practice that not all ingredients are necessarily required for success, and that the combination of ingredients is also an important consideration.

 Whilst the metaphor "active ingredients" has a reasonable level of utility, I agree with the authors that it should be applied with caution. "Ingredients" does imply a formula, and arts interventions are complex systems. The authors acknowledge the limitations of this metaphor, and that these activities may ultimately be "more than the sum of their parts" or ingredients. The INNATE framework is undoubtedly a great leap forward in specifying the active ingredients of arts in health activities, however, it may be that human experience of such complex systems still lingers somewhat elusively beyond the reach of logical frameworks and neat categorisation.

Is the work clearly and accurately presented and does it cite the current literature?

Yes

If applicable, is the statistical analysis and its interpretation appropriate?

Not applicable

Are all the source data underlying the results available to ensure full reproducibility?

Yes

Is the study design appropriate and is the work technically sound?

Yes

Are the conclusions drawn adequately supported by the results?

Yes

Are sufficient details of methods and analysis provided to allow replication by others?

Yes

Reviewer Expertise:

practitioner-researcher in singing for health and wellbeing

I confirm that I have read this submission and believe that I have an appropriate level of expertise to confirm that it is of an acceptable scientific standard.

## References

[1] : Group singing as a resource for the development of a healthy public: a study of adult group singing. *Humanities and Social Sciences Communications* .2020;7(1) : 10.1057/s41599-020-00549-0 10.1057/s41599-020-00549-0

# Response

**Published:** 2022-04-22

**Authors:** Katey Warran

**Affiliations:** University College London, UK

Thank you very much for reviewing our article. We are delighted to hear that you feel our INNATE framework is an important contribution to the field, and that our methods and results are clearly articulated. We are also pleased that you consider our implications and limitations to be discussed thoroughly.

 Based on your specific recommendations, we have added that our participants were those working in the field of arts and health in our abstract (briefly because of word count), and referenced Camlin (2020) to show wider acknowledgement of viewing the arts as complex systems. Thank you for the suggestions.

 As you note, yes, this is indeed true that some ingredients may be more or less salient in certain contexts than others, and that we have not put forward a hierarchy. Rather, we have provided a framework for identification of ingredients that can prompt reflexivity on which ingredients may be important to health outcomes. As you highlight, we discuss this in our discussion and explain that not all ingredients are required, and that users should be cautioned against feeling that every ingredient needs to be present in an activity. It remains for future research to explore which combinations of ingredients are needed to lead to specific health outcomes.

 Thank you also for your critical reflection on the use of the metaphor ‘active ingredients’, recognising that we have grappled with the limitations and benefits of using this term. We are delighted that you feel that the framework is a great leap forward.

# Peer review: Reviewer response for version 1

**DOI:** 10.21956/wellcomeopenres.19253.r47982

**Published:** 2022-01-25

**Authors:** Gunter Kreutz

**Affiliations:** 1 Department of Music, Speech and Music Lab, Carl von Ossietzky University Oldenburg, Oldenburg, Germany

The authors offer an in-depth perspective on health-related arts practices for health and wellbeing. The approach is unique as it embraces qualitative social science techniques to identify or rather unpack the multitude of facets that characterize arts interventions in this domain.

 The work suggests a very rich repository to construct working hypotheses for future investigations. The practitioners' views are highly valuable, the exclusive exploration of the supply side in arts-for-health interventions appears justified.

 What I wonder is why questions like how experiences feedback on practitioners and how they balance personal, artistic, and social demands, whether they receive, need, or miss supervision, recognition, better salaries, etc. are only scarcely addressed. Such responses would be informative about the professionalization of delivery. There is little information in the manuscript on the "64 participants", the respective Table does not mention demographics, levels of experience, training, position (full/part-time) etc.

 The target groups are talked "about", but not included in this survey. Obviously, there is a great deal of sensitivity to the clients/patients, but beyond the consideration that understanding clients/patients is important with respect to consequences for practice, not much information is given.

 The authors admit in their limitations that there is a possibility of oversimplification. However, there is also a danger of overspecification, because, without the input from the demand side, the more or less important ingredients cannot be identified. A recent study has shown that experimenter-chosen music differs radically from client-chosen music in pain patients. All arguments for a "good choice" fell apart as it was clear that the majority of pain patients seemed to need something completely different.

 I think that professionalization, training, and monitoring/supervision, are topics that may contain rather decisive information on the supply side. Missing out on those appears to me as a limitation.

Is the work clearly and accurately presented and does it cite the current literature?

Yes

If applicable, is the statistical analysis and its interpretation appropriate?

Not applicable

Are all the source data underlying the results available to ensure full reproducibility?

Partly

Is the study design appropriate and is the work technically sound?

Yes

Are the conclusions drawn adequately supported by the results?

Partly

Are sufficient details of methods and analysis provided to allow replication by others?

Yes

Reviewer Expertise:

music psychology, music and health

I confirm that I have read this submission and believe that I have an appropriate level of expertise to confirm that it is of an acceptable scientific standard, however I have significant reservations, as outlined above.

# Response

**Published:** 2022-04-22

**Authors:** Katey Warran

**Affiliations:** University College London, UK

Thank you for your review. We are delighted to hear that you feel our article offers an in-depth perspective and takes a unique approach to exploring the complexities of arts in health interventions, also facilitating future investigations. We are grateful for your critical feedback and respond to your specific comments below.

 Thank you for your feedback regarding the inclusion of practitioners’ experiences. The INNATE framework is intended to support with the design, implementation, and evaluation of arts in health activities. Given this focus on the delivery of activities (what you have described as the ‘supply side’ that you felt was justified), we feel that our inclusion of ingredient categories - which can be used to prompt reflexive processes on the more nuanced experiences of practitioners - is suitable in view of the aim of our framework. Explaining further, the worksheet is designed in a way so that the user (which could be a practitioner) reflects on the kinds of responses you have raised. For example, in the ‘activity facilitation’ section of the worksheet, the ingredient of ‘professionalization’ may prompt the user to question whether they need more supervision, or the ‘fees’ section in ‘project set-up’ category may prompt reflection on the need for better salaries. Thus, if a practitioner is affected by the different demands you mention, they will note these within our ingredient categories. Our worksheet supports with identifying these issues, rather than making normative assertions about what is the ‘right’ or ‘wrong’ way to deliver an arts in health activity. We hope that our framework will support with future research exploring how to optimise activities for improved health. We have now clarified this further in our manuscript.

 Regarding the information about our participants, we interviewed individuals based on their professional capacity, not their personal capacity. Thus, we chose not to collect demographic and individual data, instead focusing on understanding what our participants viewed as the ingredients of the activities they were involved in delivering. We were interested in the content of participants’ speech relating to their expert knowledge as generalists, rather than their individual subjectivities and personal experiences – we have now made this clearer in our manuscript. Nevertheless, we did also collect information relating to the geographic location in which our participants’ work was being delivered. We have now added this into our table to provide a richer picture of the activities that were discussed to cocreate our INNATE framework, building on your recommendation that further information would be welcome.

 In relation to your comment regarding talking ‘about’ our target group, we would like to emphasise the purpose of our research. Our aim was to create a framework to support those designing, delivering, implementing, and evaluating arts in health activities (the ‘supply side’). This aim was constructed very carefully as there are a lack of implementation tools available for those working in the field of arts and health to support with understanding active ingredients. Our intention was to provide a framework for those developing arts in health activities, rather than necessarily for those experiencing the activities themselves. Nonetheless, those engaging could work in partnership with those designing and delivering activities to use this framework. We advocate for the cocreation of arts in health activities, for example involving those with lived experience to codesign activities, where our framework could be used as part of a collaborative exercise.

 Finally, as you note, participants may perceive there to be different active ingredients than those identified in this framework, and we have now mentioned this as a limitation of our work. Whilst our framework could be used with participants to collaboratively identify active ingredients, and the remit here was to provide a tool for those developing activities, it remains for future research to explore further the ‘demand side’. There is a gap to identify research questions that are important in view of those experiencing and engaging in arts in health activities which may differ from the interests of those on the ‘supply side’, as we have explored here.
